# Nutritional markers and proteome in patients undergoing treatment for pulmonary tuberculosis differ by geographic region

**DOI:** 10.1371/journal.pone.0250586

**Published:** 2021-05-05

**Authors:** Leah G. Jarsberg, Komal Kedia, Jason Wendler, Aaron T. Wright, Paul D. Piehowski, Marina A. Gritsenko, Tujin Shi, David M. Lewinsohn, George B. Sigal, Marc H. Weiner, Richard D. Smith, Joseph Keane, Jon M. Jacobs, Payam Nahid

**Affiliations:** 1 Division of Pulmonary and Critical Care Medicine and UCSF Center for Tuberculosis, University of California San Francisco, San Francisco, California, United States of America; 2 Department of Pharmacokinetics, Pharmacodynamics & Drug Metabolism (PPDM) Merck & Co., Inc., West Point, Pennsylvania, United States of America; 3 Seattle Children’s Hospital, Seattle, Washington, United States of America; 4 Biological Sciences Division and Environmental Molecular Sciences Laboratory, Pacific Northwest National Laboratory, Richland, Washington, United States of America; 5 Voiland School of Chemical Engineering and Bioengineering, Washington State University, Pullman, Washington, United States of America; 6 Pulmonary and Critical Care Medicine, Oregon Health & Science University, Portland, Oregon, United States of America; 7 Meso Scale Diagnostics, Rockville, Maryland, United States of America; 8 University of Texas Health Science Center at San Antonio and the South Texas VAMC, San Antonio, Texas, United States of America; 9 Department of Clinical Medicine, Trinity Translational Medicine Institute, St. James’s Hospital, Dublin, Ireland; Rutgers Biomedical and Health Sciences, UNITED STATES

## Abstract

**Introduction:**

Contemporary phase 2 TB disease treatment clinical trials have found that microbiologic treatment responses differ between African versus non-African regions, the reasons for which remain unclear. Understanding host and disease phenotypes that may vary by region is important for optimizing curative treatments.

**Methods:**

We characterized clinical features and the serum proteome of phase 2 TB clinical trial participants undergoing treatment for smear positive, culture-confirmed TB, comparing host serum protein expression in clinical trial participants enrolled in African and Non-African regions. Serum samples were collected from 289 participants enrolled in the Centers for Disease Control and Prevention TBTC Study 29 (NCT00694629) at time of enrollment and at the end of the intensive phase (after 40 doses of TB treatment).

**Results:**

After a peptide level proteome analysis utilizing a unique liquid chromatography IM-MS platform (LC-IM-MS) and subsequent statistical analysis, a total of 183 core proteins demonstrated significant differences at both baseline and at week 8 timepoints between participants enrolled from African and non-African regions. The majority of the differentially expressed proteins were upregulated in participants from the African region, and included acute phase proteins, mediators of inflammation, as well as coagulation and complement pathways. Downregulated proteins in the African population were primarily linked to nutritional status and lipid metabolism pathways.

**Conclusions:**

We have identified differentially expressed nutrition and lipid pathway proteins by geographic region in TB patients undergoing treatment for pulmonary tuberculosis, which appear to be associated with differential treatment responses. Future TB clinical trials should collect expanded measures of nutritional status and further evaluate the relationship between nutrition and microbiologic treatment response.

## Introduction

Africa is home to 11% of the world’s population but carries 29% of the global burden of tuberculosis (TB) [1]. According to the World Health Organization (WHO), 84% of TB-related deaths worldwide occur in Africa [2]. It is important to understand whether there are any features of the host or phenotypes of TB disease in Africa that are unique and that may benefit from novel strategies or treatments to combat the disease more effectively.

In three contemporary, rigorous, international multicenter phase 2B clinical trials evaluating new regimens for drug-susceptible TB conducted by the Tuberculosis Trials Consortium (TBTC), significant differences in the rate of culture conversion between patients enrolled in sub-Saharan Africa versus non-African sites were consistently noted [3–6]. In TBTC Study 29 (ClinicalTrials.gov Identifier: NCT00694629), culture negativity on liquid media was lower among participants at sub-Saharan African compared with non-African sites at the end of the intensive phase of treatment, however, at the same time, negativity on solid media was higher among participants at African compared with non-African sites [4]. In TBTC Study 28 (ClinicalTrials.gov Identifier: NCT00144417) a trend in the same direction, but of lesser magnitude, was observed [4]. Comprehensive evaluations of potential causes for lower culture conversion rates were undertaken [7], and the differences in African versus non-African sites were not explained by baseline characteristics such as HIV status, severity of disease at time of enrollment, age, smoking, diabetes or race. Additionally, detailed reviews of TB laboratory procedures and quality monitoring practices were found to be within accepted practice for clinical diagnostic laboratories, and no causal issues were identified [7].

Microbial factors have also been evaluated as part of an assessment of *M*. *tuberculosis* lineage and potential impact on presentation of disease and culture conversion in response to treatment [8]. Disease presentation and response to drug treatment varied by lineage, but these associations were not statistically significant after adjustment for other variables associated with week-8 culture status. Host factors could also play a role in the differences noted in bacteriologic response rates of participants enrolled in African vs. non-African sites, and to date have not been evaluated rigorously.

We recently published a study exploring the global host proteome change in response to 8 weeks of combination therapy for drug-susceptible pulmonary TB [9]. A total of 244 proteins, mapped across numerous biological pathways, showed significant dynamic changes in response to treatment among TBTC Study 29 participants.

In the present study, we evaluated baseline protein repertoire differences between participants enrolled in African vs. non-African regions, all of whom are initiating treatment for smear-positive, culture-confirmed, drug-susceptible TB. Additionally, we sought to provide an unbiased characterization of the serum proteome in TB patients undergoing treatment, comparing serum protein signatures indicative of treatment effect by African vs. non-African region. Identifying host-related features that may underlie differential treatment response could aid in the development of new strategies and interventions that may be essential in improving TB outcomes and reducing TB mortality in Africa.

## Materials and methods

### Ethics statement

Study 29 was approved by both CDC and local institutional review boards, and written informed consent was obtained from all study participants for collection of serum for TB-related research. The institutional review board at University of California, San Francisco approved this biomarker study (UCSF IRB Number: 12–10360).

### Study population

TBTC Study 29 (ClinicalTrials.gov Identifier NCT00694629) is a prospective, multicenter, open-label Phase 2 clinical trial comparing the antimicrobial activity and safety of a standard daily regimen containing rifampin, to that of an experimental regimen with daily rifapentine (10 mg/kg/dose), both given with isoniazid, pyrazinamide and ethambutol to adults with smear positive, culture-confirmed pulmonary TB. Participants represented in this analysis were enrolled at 15 TBTC sites (10 in North America, 3 in South Africa, and 1 each in Uganda and Spain). Adults (age ≥18 years) with suspected pulmonary tuberculosis and acid-fast bacilli (AFB) in a sputum specimen were eligible. The primary efficacy endpoint of the trial was the proportion of patients, by regimen, having negative sputum cultures on liquid and solid media at completion of 8 weeks (40 doses) of treatment. All TB treatment was administered 5 days/week for 8 weeks per protocol on an empty stomach and directly observed. All participants underwent HIV testing. Cultures were performed using both Lowenstein-Jensen (LJ) solid media (inoculum volume, 0.2 mL) and BACTEC mycobacterial growth indicator tube (MGIT, Becton Dickinson and Co, Franklin Lakes, New Jersey) liquid media (inoculum volume, 0.5 mL) with the MGIT 960 system, and assessed for presence of *M*. *tuberculosis*. Inclusion criteria were Karnofsky score ≥60; serum alanine aminotransferase (ALT) ≤3 times upper limit of normal (ULN), total bilirubin ≤2.5 times ULN, and creatinine ≤2 times ULN; hemoglobin ≥7.0 g/dL; platelets ≥100 000/mm3; negative pregnancy test for women; ≤5 days of multidrug antituberculosis treatment in the preceding 6 months; and ≤7 days of fluoroquinolone treatment in the preceding 30 days. Exclusion criteria were pregnancy or breast-feeding; weight <40 kg; central nervous system tuberculosis; pulmonary silicosis; allergy, intolerance, or contra-indicating condition to any of the study drugs; current therapy or therapy planned in the subsequent 8 weeks with antiretroviral medications, cyclosporine, or tacrolimus; and initial sputum cultures negative for Mycobacterium tuberculosis or with growth of an Mycobacterium tuberculosis strain resistant to rifampin, isoniazid, or pyrazinamide. Additional information regarding the design, conduct, protocol correct criteria, and results of TBTC Study 29 has been published [4].

### Selection of participants

A subset of 289 consecutively enrolled protocol-correct participants from TBTC Study 29 [9] for whom serum samples were available, from clinical trial sites in North America, Spain, South Africa, and Uganda were included in this study. Detailed demographic, clinical, radiographic and microbiologic data were collected using TBTC-developed case report forms as part of the parent clinical trial. Given the exploratory biomarker discovery objectives of this project, 270 HIV-uninfected participants with paired samples were included in the current analyses. The paired datasets utilized for this study were stratified based on site and region into an African cohort comprised of samples collected from study participants enrolled in multiple sites within Uganda and South Africa, and a non-African cohort comprised of samples from participants in North America and Spain.

### Sample processing and analysis

Details of processing and analysis of 540 samples are described elsewhere [9]. Briefly, samples included in this study were collected at baseline and after 8 weeks of therapy from consenting participants enrolled in TBTC Study 29 clinical trial. Individual human serum samples were partitioned and depleted of 14 specific highly abundant proteins using a ProteomeLabTM 12.7 × 79.0-mm human IgY14 LC10 affinity LC column (Beckman Coulter, Fullerton, CA). Further processing of the flow-through portion of the depletion was as previously described [9] including analysis of digested peptides on the ion mobility (IM) separation platform coupled with an Agilent 6224 TOF MS [10].

### Selected Reaction Monitoring (SRM)

SRM was performed on a random subset of 96 baseline serum samples that represented a proportional sample from relevant regions and subgroups, and included 50 samples from Africa, (25 each from South Africa and Uganda), 13 from Spain, and 33 from North America including 17 from self-reported Black and 16 non-Black race participants. Crude heavy peptides labeled with 13C/15N on C-terminal lysine and arginine were purchased from New England peptides (Gardner, MA). Trypsin digested samples that had been stored at -80°C until use were processed as previously described [11]. As SRM samples were not processed using the IgY14 depletion protocol, the SRM results are removed from any possible quantitative bias introduced from this immunoaffinity depletion in comparison across different population demographics. For each sample the digested peptides were diluted to 0.2 μg/μL containing standards at a final concentration of 250 fmol/μL for 11 protein standards and 500 fmol/μL for 12 proteins. All the samples were analyzed with a nanoACQUITY UPLC^®^ system (Waters Cooperation, Milford, MA) coupled online to a TSQ Vantage triple quadrupole mass spectrometer (Thermo Scientific, San Jose, CA). The LC-SRM platform was configured and utilized as previously described [12].

SRM data acquired on the TSQ Vantage were analyzed using Skyline software [13]. Peak detection and integration were determined based on retention time and the relative SRM peak intensity ratios across multiple transitions between light peptide and heavy peptide standards [14]. All the data were manually inspected to ensure correct peak assignment and peak boundaries. The peak area ratios of endogenous light peptides and their heavy isotope-labeled internal standards (i.e., L/H peak area ratios) were then automatically calculated by Skyline, and the average peak area ratios from all the transitions were used for quantitative analysis of the samples. For targets that had more than one surrogate peptide, correlation graphs were plotted to verify a strong correlation and ultimately the peptide that had the most sensitive response was selected for obtaining quantitative values.

### Statistical analysis

Categorical clinical and demographic characteristics were compared by site using Chi squared tests; medians were compared by global region using the Wilcoxon rank-sum test. Peptide values were log2 transformed and normalized for occurrence filtering, outlier removal, and merger into protein level quantitative values as previously described [10]. Each protein was individually tested for univariate association with clinical and demographic variables using linear regression with protein expression as the continuous outcome. Significance levels were stratified into p-value bins to compare the relative impact of patient characteristics on the global proteome. Protein expression was compared by African/non-African site using ANOVA adjusted for lung cavitation, race, and low BMI as a random effect. Cox proportional hazards regression was used to assess interactions between African/non-African site and markers of nutritional status as predictors of time to culture conversion on liquid media. Correlations between region and nutritional status (weight, bmi, and apolipoproteins APOA1, APOA2, APOA4, APOB, APOC1, APOC2, APOC3, APOH) were assessed using the Spearman rank test where p < 0.01 was considered significant. All statistics were calculated using the R computing environment (www.R-project.org, 2018). Protein pathway mapping and enrichment was done using the functional annotation tool DAVID (david.ncifcrf.gov). Heat map visualizations were created using the in-house developed program Inferno, a successor to the program DAnTE [15].

## Results

Participants’ demographics, clinical characteristics, and treatment outcomes differed by study site. Patients at African sites were younger and had lower body mass index (BMI) than those at non-African sites (median age was 27.3 vs. 44.6 years, and median BMI was 19 vs. 22 kg/m^2^, respectively). At baseline, 40.8% of African participants were underweight (defined as BMI ≤18.5) compared to 14.6% of non-Africans. More Africans presented with fever and productive cough (fever: 68.0% vs. 47.5% and productive cough: 95.9% vs. 79.7%, respectively). Africans had somewhat higher platelet count at baseline than non-Africans (403 vs. 373 10^9^/L, respectively). Presenting with AFB smear classification 3–4+ at baseline was similar across the two groups, but Africans had shorter median days to detection on MGIT960 than non-Africans (6.7 vs. 8.3 days, respectively). Rates of cavitation were similar in both groups, but more Africans had cavities >4cm (50.3% vs. 25.2%, respectively). Fewer Africans achieved stable culture conversion by week 8, the end of the intensive phase of therapy (53.7% vs. 72.5%, respectively), and more were still culture positive at week 12 (20.4% vs. 9.2%, respectively). African participants on average took longer to achieve stable culture conversion on liquid media (56 vs. 42 days, respectively), but days to conversion on solid media were similar (42 vs. 41.5 days, respectively). Africans and non-Africans gained weight at similar rates during the first 8 weeks of treatment, but a higher proportion of Africans remained underweight at week 8 (28.3% vs. 11.4%, respectively) (Table 1).

**Table 1:** Participant characteristics by study site.

	African study site n (%)	Non-African study site n (%)	p value
N	147	123	
**Demographics**			
Median age, years (IQR)	27.3 (23.3–33.6)	44.6 (31.2–53.9)	<0.001
Female	39 (26.5)	42 (34.1)	0.220
Median weight, kg (IQR)	53.2 (48.3–58.0)	60.8 (54.9–68.0)	<0.001
Median body mass index, kg/m^2^ (IQR)	19 (17–21)	22 (20–24)	<0.001
Underweight (BMI < 18.5)	60 (40.8)	18 (14.6)	<0.001
Highest educational level:			<0.001
Eighth grade or less	57 (39.0)	37 (30.1)	
>Eighth and <12^th^ grade	52 (35.6)	30 (24.4)	
Completed 12^th^ grade	29 (19.9)	26 (21.1)	
Some college or more	8 (5.5)	30 (24.4)	
**Baseline lab values**			
Median hemoglobin, g/dL (IQR)	12.5 (11.3–13.3)	12.4 (11.6–13.8)	0.350
Median platelets x10^3^/mm^3^ (IQR)	403 (330–504)	373 (292–461)	0.014
Median white blood cells x10^3^/mm^3^ (IQR)	7.7 (6.6–9.9)	8.5 (6.8–10.0)	0.272
AST > upper limit of normal	16 (10.9)	13 (10.6)	1.000
**Baseline clinical factors**			
Smoking history	48 (32.7)	64 (52.0)	0.001
Drug use	13 (8.8)	11 (9.0)	1.000
Alcohol abuse	7 (4.8)	11 (9.0)	0.252
HIV positive	0	0	
TB symptoms:			
Fever	100 (68.0)	57 (47.5)	0.001
Sweats	90 (61.2)	64 (52.0)	0.162
Productive cough	141 (95.9)	98 (79.7)	<0.001
AFB smear grade 3–4	105 (71.4)	73 (60.8)	0.089
Cavities present	101 (68.7)	82 (66.7)	0.820
Chest x-ray classification:			<0.001
No cavities	45 (30.6)	49 (39.8)	
Cavities <4cm	28 (19.0)	43 (35.0)	
Cavities >4cm	74 (50.3)	31 (25.2)	
Median days to detection on MGIT960 (IQR)	6.67 (5.04–8.50) [n = 145]	8.29 (6.04–11.1) [n = 105]	<0.001
**Treatment factors**			
Treatment arm			0.460
HP_10_ZE (rifapentine)	83 (56.5)	63 (51.2)	
HR_10_ZE (rifampin)	64 (43.5)	60 (48.8)	
Received pre-study anti-TB doses	59 (40.1)	107 (87.0)	<0.001
Median n pre-study doses (IQR) [Range]	4 (2–4) [1–11]	4 (2–5) [1–12]	0.708
**Treatment response**			
Week 8 AFB smear grade 3–4	9 (6.1)	5 (4.1)	0.650
Week 8 median BMI (IQR)	20 (18–21)	23 (21–25)	<0.001
Week 8 underweight (BMI <18)	43 (29.3)	14 (11.4)	<0.001
Week 8 median BMI change (IQR)	1 (0–1)	1 (0–2)	0.949
Week 8 median weight change, kg (IQR)	0.29 (0.09–0.49)	0.23 (0.05–0.51)	0.694
Week 8 stable conversion on solid & liquid media*	79 (53.7)	87 (72.5)	0.002
Week 8 median minimum days to detection on MGIT960 (IQR)	20.7 (15.6–24.5) [n = 65]	21.5 (19.0–26.5) [n = 25]	0.114
Median days to stable culture conversion on liquid media (IQR)	56 (56–84)	42 (28–56)	<0.001
Median days to stable culture conversion on solid media (IQR)	42 (28–56)	41.5 (17–55)	0.005
Week 12 culture positive	30 (20.4)	11 (9.2)	0.018

*Three non-African cases were missing data for solid media conversion but successfully converted at 28, 43, and 56 days on liquid media.

Abbreviations: IQR = inter-quartile range, BMI = body mass index, AST = aspartate aminotransferase, AFB = acid fast bacilli.

### Human proteome differs consistently by African and non-African region: A region-specific proteomic signature

A total of 10,137 peptides corresponding to 872 proteins were identified and quantified from 270 participants’ paired samples collected at baseline and after 8 weeks of treatment [9]. Among the demographic and clinical variables reported (Table 1), the most significant driver of protein differences was enrollment region, specifically African or non-African site (S1 Fig).

**Fig 1:**
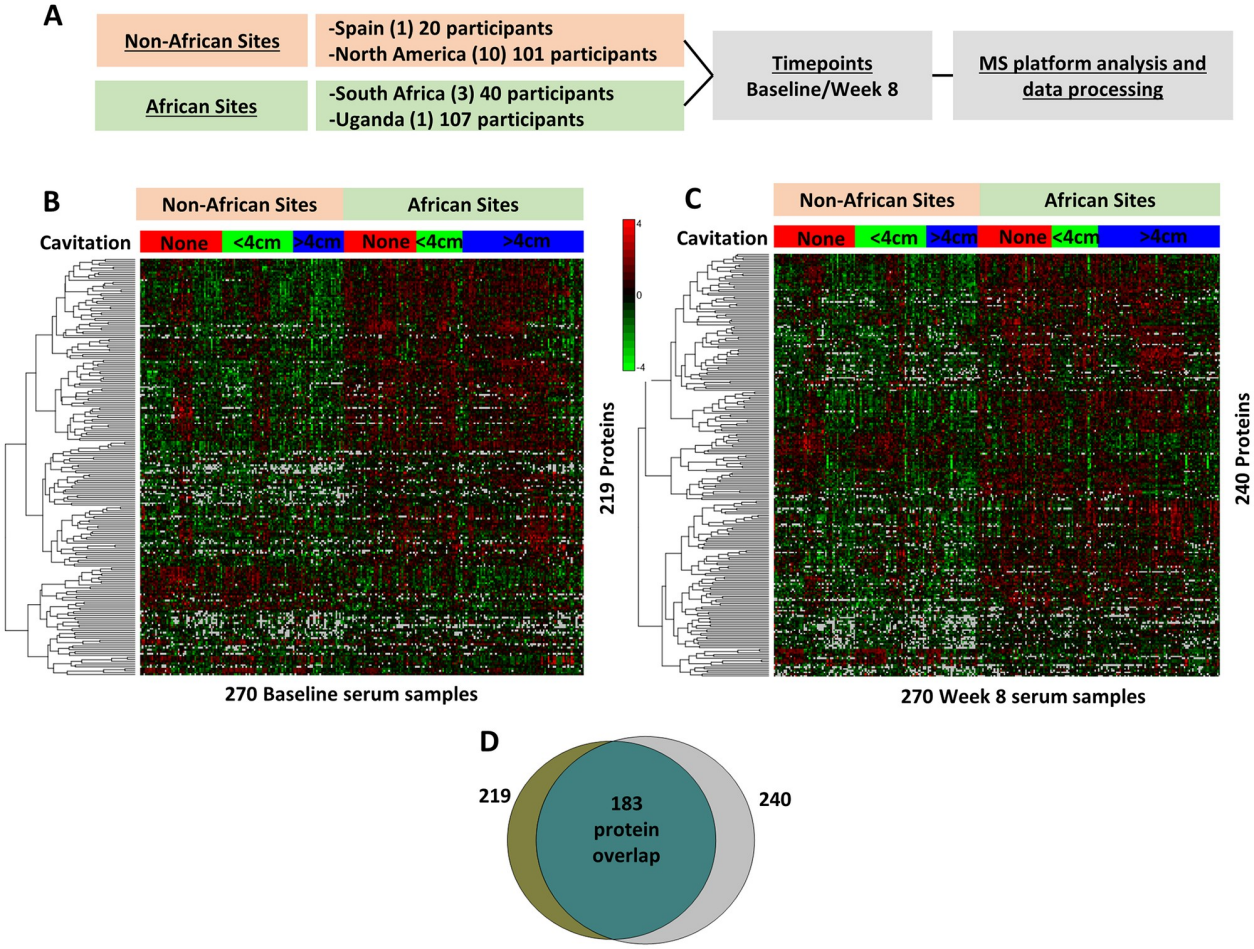
Comparison of African and non-African serum plasma proteome data. **A)** Schematic view of study sites and participants. Serum proteome data was compared at **B)** baseline and **C)** after eight weeks of treatment across 270 patients to determine the significant proteins, p value <0.05, that differentiate African versus Non-African patients. Disease severity is representing by stratification by cavitary size. 219 and 240 proteins were identified respectively from these time points which resulted in an overlap of **D)** 183 proteins, which forms a core protein signature of African/Non-African differentiation independent of treatment.

We identified 219 proteins at baseline (Fig 1B) and 240 proteins at week 8 (Fig 1C) whose abundance was significantly different in samples from African vs. non-African sites (S1 Table). We expected that treatment after 8 weeks would affect the protein abundance differential, or close the gap, between underweight African patients with higher burden of TB disease and their non-African counterparts, but many of the same proteins that were different by African site at baseline continued to be significantly different at week 8 (Fig 1D). In total, there were a core set of 183 proteins that differed by site at both time points, representing an intrinsic serum proteome signature that differentiated between African and non-African TB study participants independent of treatment (Fig 1). This discrimination pattern at both baseline and week 8 can also be observed in principal component analysis (PCA) plots based upon the correlation of the quantified serum protein values, showing delineation between African vs. non-African participants (S2 Fig). Proteins that were significantly up- or downregulated at only one time point were likely those influenced by treatment or were more variable in abundance and did not achieve the significance threshold in both comparisons.

**Fig 2:**
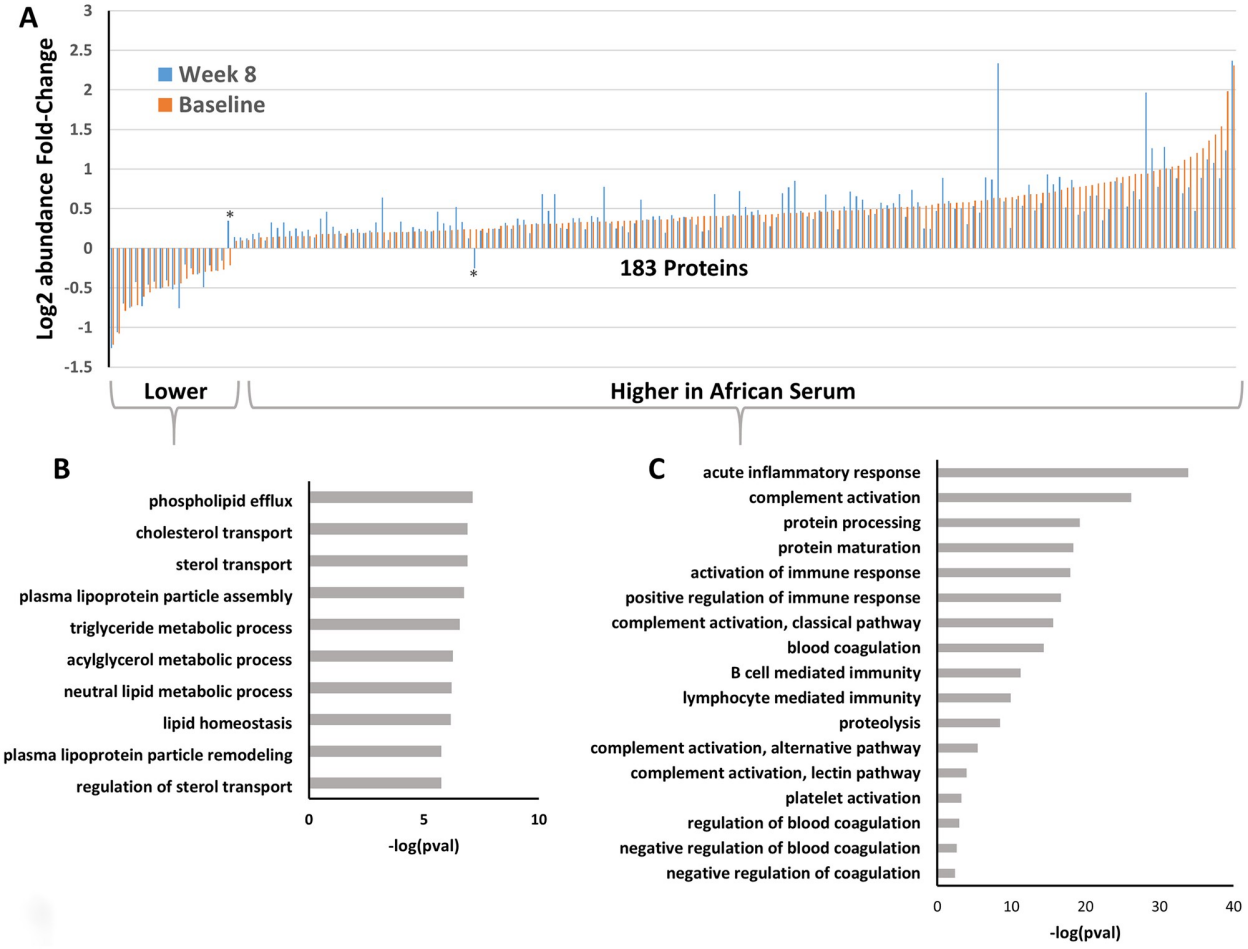
Characterization of the 183 African/non-African discriminatory core protein signature. Shown in **A)** is the fold change difference in abundance between African and non-African TB patient population for each of the 183 proteins in rank order. Negative, lower, fold change is in reference to the African population. Asterisks mark two proteins SHBG and TIMP1, which change directionality between baseline and week 8 results, where all other proteins show similar directionally of change between these two time point datasets. B) Gene Oncology (GO) annotations with Bonferroni adjusted p-values generated via DAVID show those pathways downregulated in African populations. C) Protein pathways upregulated in African populations.

Almost all the signature proteins (181/183) were consistently differentially upregulated or downregulated by African/non-African site at both time points; the two exceptions were proteins TIMP-1 and SHBG (denoted by asterisks, Fig 2A). Proteins downregulated in African participants show a clearly different pathway annotation (lipid transport and metabolism) (Fig 2B) compared to those upregulated in Africans (inflammatory, acute phase, complement, and immunity pathways) (Fig 2C).

Validation of upregulated and downregulated pathways by African/non-African site was performed for a set of representative proteins (CRP, A1AG1, TTHY, APOA1) using an orthogonal peptide sequence specific targeted selective reaction monitoring (SRM) MS analysis approach (S3 Fig). Results confirmed the quantitative findings of our global analysis, showing significant upregulation of inflammatory mediators and downregulation of lipid and hormone metabolism proteins.

### Signature host proteomic differences by African/non-African site are not explained by differences in disease severity

**Fig 3:**
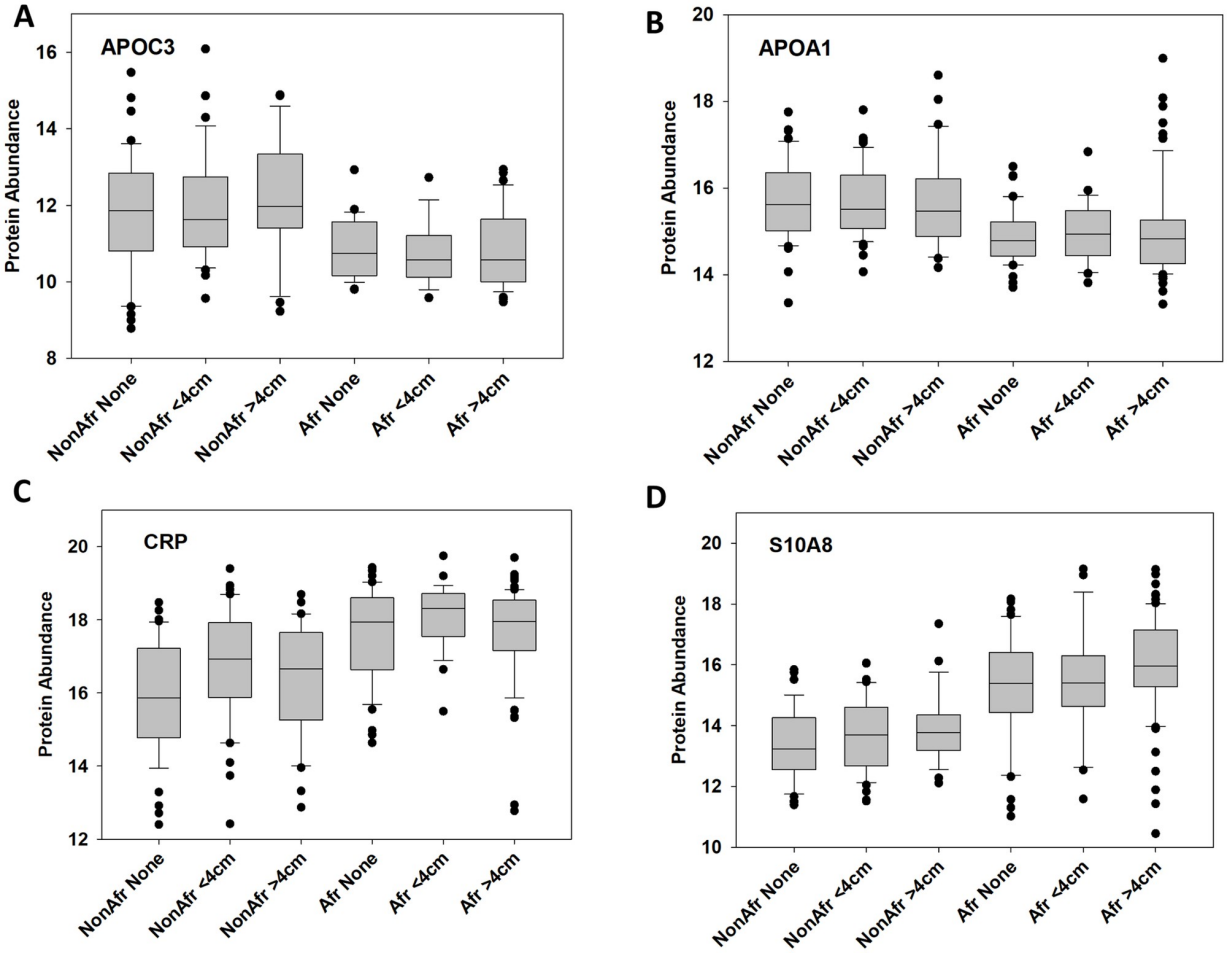
Boxplot of representative proteins across cavitary size. **A-D)** Boxplot representation of quantitative global MS data from baseline values for two proteins representative of lipid and nutrient transport downregulation within the African cohort and two proteins representing acute inflammatory activation upregulated with the African cohort, all stratified across cavitary size representing initial disease severity.

Previous studies have determined that baseline disease severity by itself did not explain poor outcome in African populations [7]. Nevertheless, we tested the effect of disease severity on the 183 signature proteins, stratifying by African site and baseline cavity size, a key indicator of disease severity at baseline. We found that for the vast majority of proteins, there was no gradient of protein abundance by cavity size within each cohort; instead, African patients with cavities >4cm had median abundance very close to that of other Africans regardless of their cavitation status, and were very different from non-Africans with cavities >4cm. Fig 3 shows the stratified African/non-African differences in a set of representative proteins (results for remaining proteins are shown in S4 Fig). Results suggest that African/non-African proteomic differences are independent of disease severity.

### Downregulated proteins indicate that Africans with TB exhibit less nourishment throughout 8 weeks

**Fig 4:**
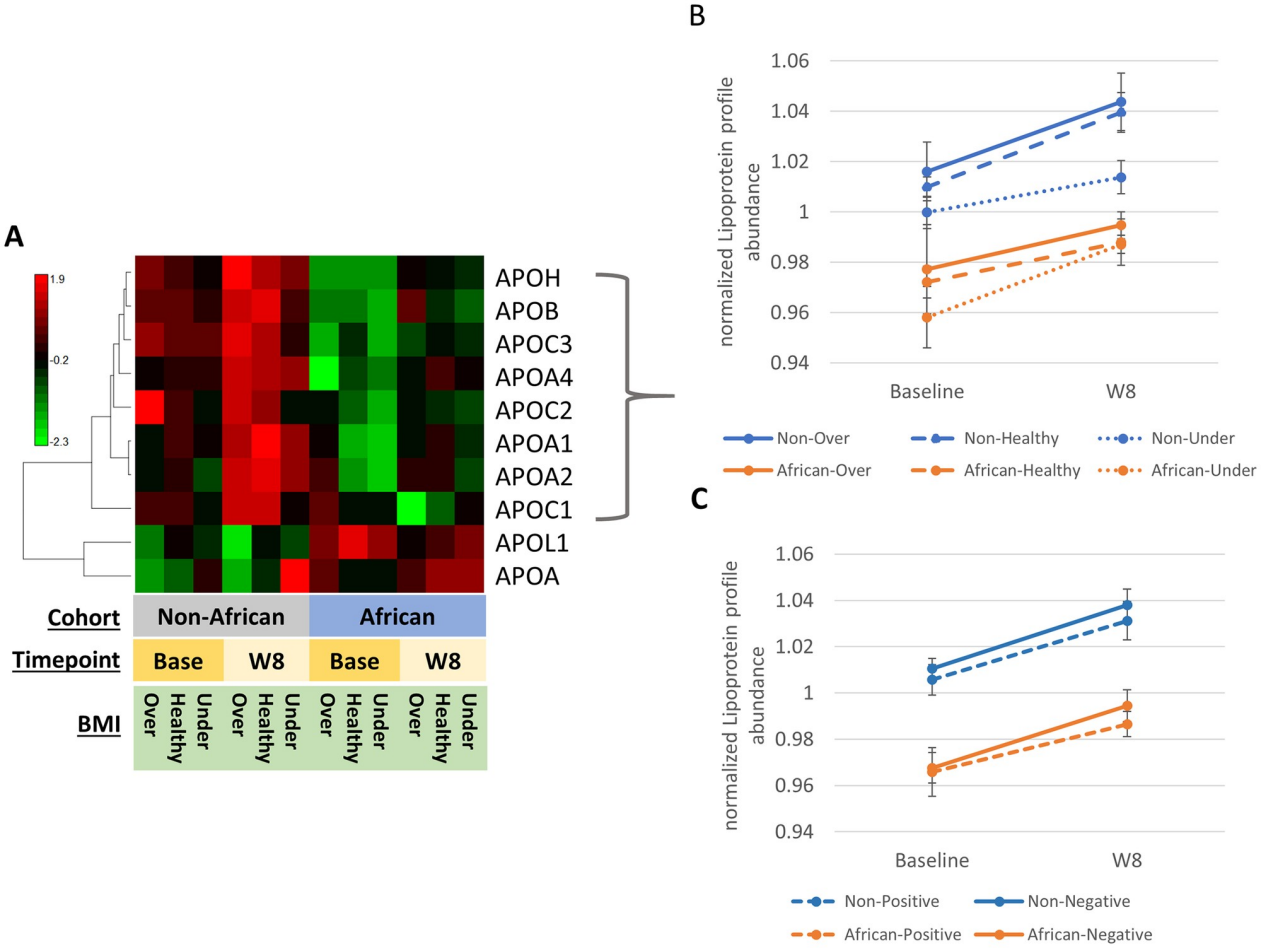
Comparison of apolipoprotein plasma signature with BMI and culture status. **A)** Heatmap, Pearson correlation, of all apolipoproteins encompassed within the African TB patient discriminatory protein signature stratified across cohort, timepoint, and BMI. Overweight >25 BMI, Healthy weight between 18.5 and 24.9 BMI, Underweight, <18.5 BMI. **B)** Graph of the apolipoprotein signature from the 8 proteins downregulated in the African cohort, stratified across BMI range. **C)** Graph of the apolipoprotein signature stratified across 8 week culture conversion status. **B)** and **C)** Values represent median protein abundances from a scaled normalization within each protein across time point and cohort. Error bars represent the standard deviation across the median protein values.

Proteins related to lipid metabolism and transport were downregulated at both time points in the African cohort (Fig 2B), suggesting to us that nutritional status might have a role in driving poor outcomes in this population. We compared lipoprotein expression with patient BMI. Study participants enrolled in Africa had lower median BMI at baseline (19 vs. 22 respectively, p<0.001), with a larger percentage of underweight participants compared to non-Africans (40.8% vs. 14.6% respectively, p<0.001). As may be expected, BMI increased in both populations upon treatment (median weight gain of 0.29kg vs. 0.23kg respectively, p = 0.694), however, a substantial gap remained by regions of enrollment even after 8 weeks of intensive phase treatment (29.3% vs. 11.4% undernourished respectively, p<0.001, Table 1). Fig 4B shows all the apolipoproteins from the protein signature, stratified across timepoint and standard BMI categories, overweight (>24.5), healthy (<24/>18.5), and underweight (<18.5). Both cohorts showed apolipoprotein upregulation after treatment, but protein abundance in the African cohort remained well below that of the non-Africans (Fig 4B). Further, Fig 4C shows the same lipoprotein signature stratified across positive culture status on solid or liquid media at 8 weeks. Results show again the large gap between African and non-African cohorts, as well as positive culture status patients consistently lagging behind in signature recovery for both groups, with African culture positive patients increasing the slowest towards lipoprotein recovery upon treatment, implying an association between culture status outcome and nutritional status, particularly at week 8. Tests for interactions between region and measures of nutritional status (specifically weight, BMI, and 8 apolipoproteins at baseline and week 8) as predictors of time to culture conversion found no discernible interactions (all p > 0.05), even when adjusted for disease severity markers such as baseline cavities >4cm, AFB smear 3+, and other factors such as age, sex, and baseline platelet count (data not shown). Rather, African region was highly correlated with nutritional markers: 19 of 20 tests showed p < 0.01, with a median Spearman *rho* = –0.38, ranging from –0.57 to –0.19. The absence of interaction suggests that nutritional factors associated with slower time to conversion have the same effect in African and non-African populations; consistent correlation suggests that African region may be a surrogate for nutritional status in these analyses.

Only 2 apolipoproteins were more abundant in the African cohort for both timepoints, apolipoprotein(a) (APOA) and Apolipoprotein L1 (APOL1) (Fig 4A), both known to be differentially regulated in African populations. APOA is known to have higher serum abundances in populations of African descent [16–18], while circulating APOL1 is known to confer resistance to African trypanosomiasis (sleeping sickness) [19, 20], primarily through presence of a G1 or G2 sequence variant, though presence of these variants also increases the risk of kidney disease [21]. The G1 and G2 variants were not included in our database searching, and the APOL1 protein abundance reported was based on the detection and quantification of peptides in common across all cohorts yet results still showed a decisive increase in APOL1 abundance in the African cohort.

### Upregulated proteins indicate that Africans have more inflammation

**Fig 5:**
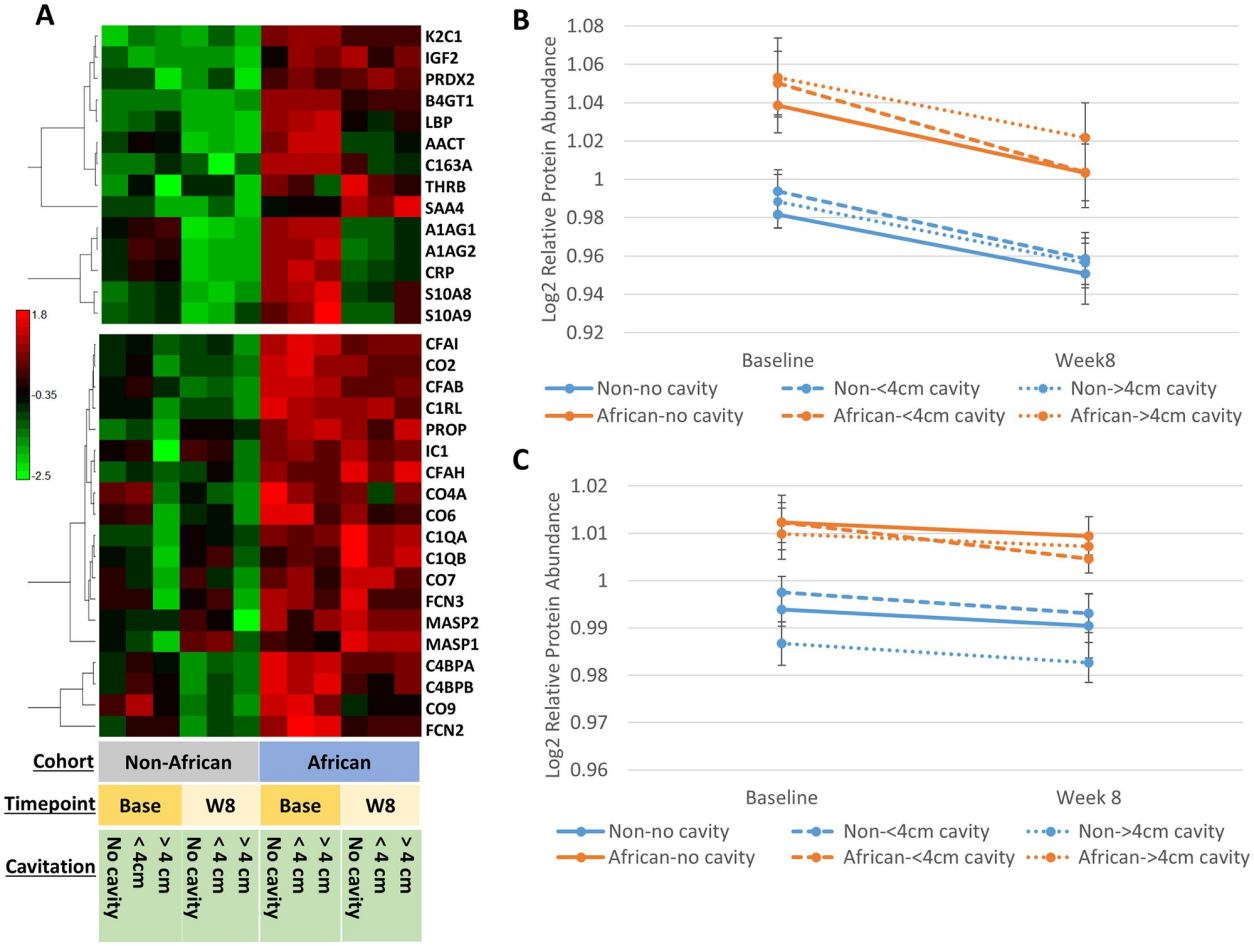
Comparison of inflammatory and complement activation plasma signature. **A)** Heatmap, Pearson correlation, of proteins found in the acute inflammatory response and complement activation pathways from Fig 2C, with redundancy removed. Results are stratified across cohort, timepoint, and disease severity as determined by cavitary size by baseline chest radiograph. **B)** Graph of the composite (14 proteins from panel **A**) acute inflammatory protein signature across timepoint and disease severity **C)** Graph of the composite (19 proteins from panel **A**) complement activation protein signature across timepoint and disease severity. Values in panel **B)** and **C)** represent median protein abundances from a scaled normalization within each protein across time point and cohort. Error bars represent the standard deviation across the median protein values.

The largest group of proteins upregulated in African vs. non-African study participants were related to inflammatory response, immune activation, and complement components (Fig 2C). Fig 5 provides a visual breakdown of the acute inflammatory response and complement activation proteins, stratified by lung cavitation and compared across time points. Results show clear separation between the African and non-African patients, regardless of cavitation severity. Both the inflammatory response (Fig 5B) and the complement activation signatures (Fig 5C) are downregulated after 8 weeks of treatment. The slope of change is greater among the inflammation-related proteins however, the African cohort expresses a higher abundance of these proteins throughout all timepoints and cavitation severity.

### Underexpressed RET4 indicates vitamin A deficiency in the African cohort

**Fig 6:**
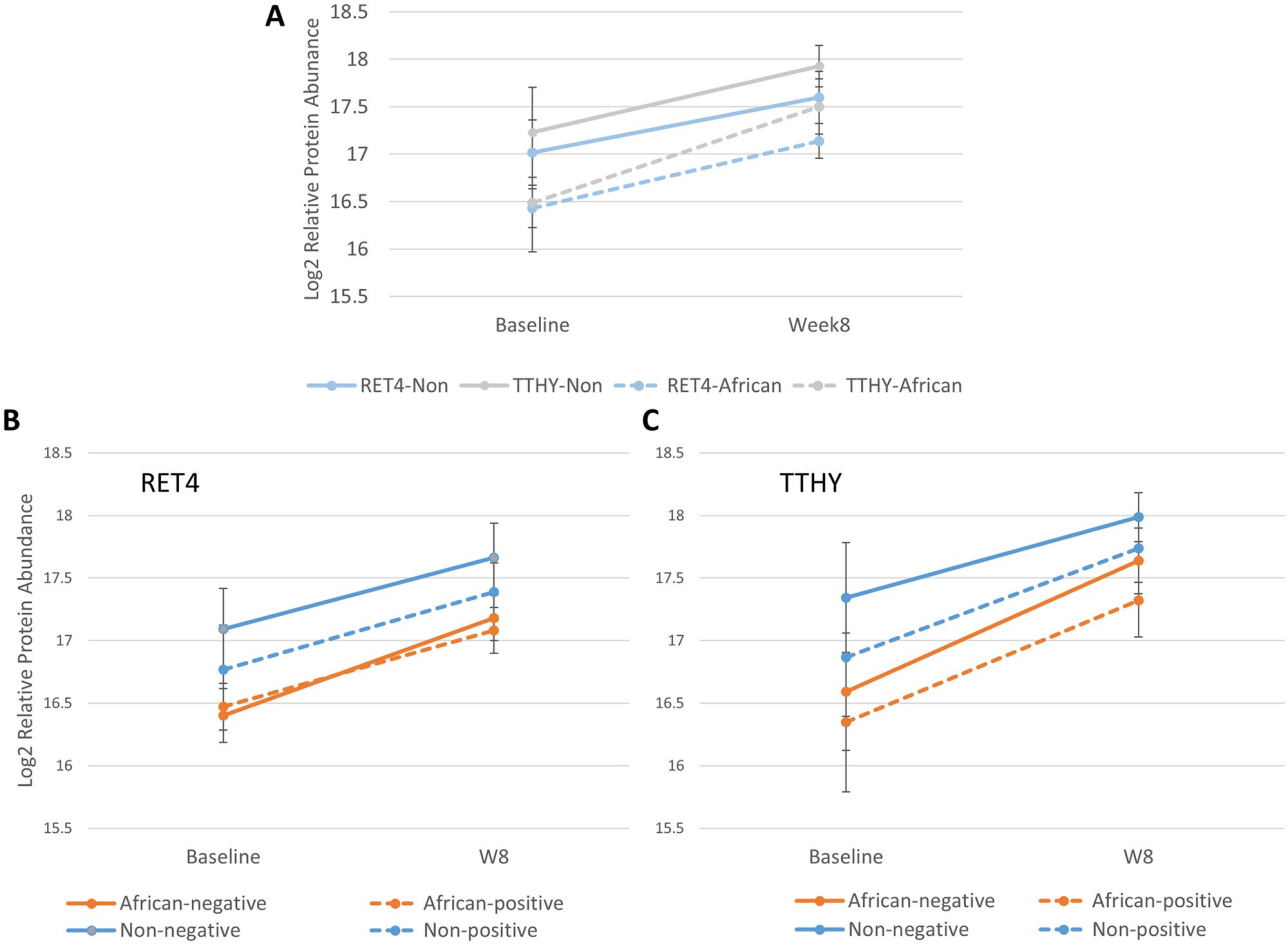
Comparing retinol binding protein and transthyretin abundances to understand inflammation and vitamin A deficiency in African TB patients. **A)** graph combining Retinal Binding Protein (RET4/RBP) and Transthyretin (TTHY) abundance changes between cohorts. **B)** Graph of RET4 only, stratified across both cohorts and patient culture status (positive encompasses both liquid and solid) at 8 weeks as a measure of outcome. **C)** Graph of TTHY only, stratified across both cohorts and patient culture status. Error bars for all panels represents the variance across patient population for each protein and time point.

Since we found that the African cohort could be differentiated by proteins generally related to nutritional and inflammatory pathways, we further investigated specific proteins that might shed light on why Africans have poorer TB outcomes. Retinol-binding protein (RBP/RET4) and Transthyretin (TTHY) were both proteins in the proteomic signature under-expressed in the African patients. Both of these proteins are known to be suppressed in infection/inflammatory conditions [22]. RET4 is also an effective measure of vitamin A status since RET4 acts as a retinol transporter and >95% of total retinol binding occurs with RET4 in plasma, yielding a close correlation between RET4 secretion and vitamin A abundance [23, 24]. Low RET4 levels in the African population indicate vitamin A deficiency in this population. However inflammation is also known to reduce RET4 levels in circulation [24]. To understand if RET4 is reduced due to inflammation or vitamin A deficiency, we used TTHY as a benchmark of inflammation. Previous studies have been able to identify vitamin A deficiency status within an inflammatory condition [25] by comparing both TTHY and RBP protein level changes upon treatment. When we graphed RET4 and TTHY plasma abundances and stratified by African/ non-African site, we see that TTHY rebounds more quickly between baseline and week 8 than RET4, particularly within the African population as inflammation is resolved by intensive phase treatment (Fig 6A). When we compared TB treatment outcome, defined as positive culture on solid or liquid media at 8 weeks, we saw clear separation in TTHY levels in both cohorts by culture status, but separation in RET4 values only occurred in the non-African cohort, with low RET4 values (indicating low vitamin A levels) appearing to be intrinsic to the African population, irrespective of culture status at 8 weeks.

## Discussion

Numerous phase 2 and 3 trials have found that culture conversion in participants from sub-Saharan African sites are delayed as compared to participants enrolled from sites in the Americas and Europe [3, 6]. In this study, we have shown that there are regional differences in the serum proteome that distinguish participants enrolled in African versus non-African sites. Differential expression occurred by region in a core set of 183 proteins both before and after 8 weeks of directly observed, intensive phase therapy. Proteins differentially upregulated in the African cohort were primarily markers of inflammatory response including acute phase reactants as well as proteins participating in coagulation and complement cascade. Proteins that were consistently downregulated in African participants were primarily markers of lipid transport, metabolism and hemostasis, and proteins indicative of nutritional status. More specifically, lipid mediators including APOC3, APOC2, APOA1, APOA2, APOA4, APOH, and APOB were universally under expressed in African TB patients’ serum. These proteins are the primary components in VLDL, LDL, and HDL compositions, including mediators of phospholipid binding (APOH), and cholesterol transport (APOA1). Other nutrient regulatory transport proteins found in lower abundance include RARR2, a broadly active adipocyte regulatory protein; RET4, retinol (vitamin A) transport protein; and TTHY, another broadly active hormone-binding, retinol transport, complex forming protein. RET4 and TTHY have been identified as possible markers of culture status, and predictive of future culture conversion [9]. Though both cohorts improved circulation of lipid and nutrient markers upon treatment, African participants remained well below their counterparts from non-African region sites. The high correlation and absence of interaction between region and nutritional markers suggests that undernutrition, though common in the African cohort studied here, is itself the cause of poor treatment outcome.

Undernutrition is known to impact TB outcomes, specifically by delaying bacteriologic sterilization [26] and increasing TB mortality and relapse [27–29]. Undernutrition and malnutrition have been shown to compromise host immune function [30–32], inhibit anti-TB drug absorption [33], reduce host appetite, protein stores and available energy to fight TB infection [34, 35], and are associated with non-adherence to treatment [36].

Since 2015, world hunger has been rising in tandem with international economic downturns [37]. Undernutrition and food insecurity are highest where rates of TB are high: the greatest incidence of hunger is in sub-Saharan Africa, where 20% of the population are undernourished [37]. WHO recognizes undernutrition as an important condition that affects TB treatment outcomes [38] and recommends using their 2013 guidelines for nutritional assessment and food supplementation when assessing TB patients [39], but dietary specifics are lacking due to a dearth of scientific evidence. To date, four trials have tested the effect of food supplements on TB treatment success [40–43]. Though small and underpowered, all four studies found that food incentives improved TB outcomes, most often accelerating bacteriologic sterilization as measured by sputum smear or culture conversion.

Our study has limitations. Based on clinical characteristics collected at baseline, the African participants in our study on average had more severe TB disease and lower BMI than non-Africans. Advanced TB and undernutrition both engender inflammatory processes which affect proteomic biomarkers. The parent trial was not designed to support deep analyses of nutritional status, inflammation as an epiphenomenon, or proteomic differences by region, so we were unable to assess the true magnitude of undernutrition and inflammation vs. African genetics and way of life on the proteomic signature. Finally, the parent clinical trial protocol limited pre-randomization TB treatment to no more than 5 doses, and we found that participants enrolled in non-African settings were proportionally more likely to have received a median of 4 pre-treatment doses. Whereas these pre-randomization doses may complicate interpretation, our finding that the regional differences in the proteomic signatures, including indicators of nutritional status, did not resolve upon completing 56 doses of intensive phase treatment, these initial doses (median 4 doses) are unlikely to have significantly impacted our results.

In summary, we have identified differentially expressed proteins by region, particularly in relation to nutrition and lipid pathways, which appear to be associated with microbiologic treatment responses. Measures of nutritional status should be included in TB clinical trials, and interventions for enhancing nutrition, in particular macronutrient supplementation and food [44], should be studied as these proteomic evaluations suggest an association between indicators of nutritional status and TB treatment effect.

## Supporting information

S1 Fig Identification of critical co-variates of serum proteome data. Univariate ANOVA analysis showing the number of proteins which are deemed significantly differential for each clinical covariate at various adjusted p value (q-value) levels. Noted is the most differential covariates of region, African status, and race (all interrelated), which is maintained through 8 weeks of treatment.
(TIF)

S2 Fig African stratification plot. Principle Components Analysis (PCA) plot showing stratification of all TB patients based on African/non-African enrollment region. Blue representing African patients, and red representing non-African patients.
(TIF)

S3 Fig SRM validation studies. Boxplot representation of Selective Reaction Monitoring (SRM) MS data of specific differential proteins to validate the previous global quantitative data. The peptide sequence which was used as the accurate quantitative internal standard is given for each protein. Two-tailed t-test p-values are provided for each comparison, which all show significance at the <0.05 confidence level.
(TIF)

S4 Fig Boxplot of additional representative proteins driving upregulation of serum pathways. **A-C)** Boxplot representation of quantitative global MS data for three additional relevant proteins representative of inflammatory, host response, and immune activation showing both up and down regulation within the African cohort stratified by cavitary size.
(TIF)

S1 Table (XLSX)
