# Correction: Nutritional markers and proteome in patients undergoing treatment for pulmonary tuberculosis differ by geographic region

**DOI:** 10.1371/journal.pone.0331721

**Published:** 2025-09-04

**Authors:** Leah G. Jarlsberg, Komal Kedia, Jason Wendler, Aaron T. Wright, Paul D. Piehowski, Marina A. Gritsenko, Tujin Shi, David M. Lewinsohn, George B. Sigal, Marc H. Weiner, Richard D. Smith, Joseph Keane, Jon M. Jacobs, Payam Nahid

The first author’s name is spelled incorrectly. The correct name is Leah G. Jarlsberg. The correct citation is: Jarlsberg LG, Kedia K, Wendler J, Wright AT, Piehowski PD, Gritsenko MA, et al. (2021) Nutritional markers and proteome in patients undergoing treatment for pulmonary tuberculosis differ by geographic region. PLoS ONE 16(5): e0250586. https://doi.org/10.1371/journal.pone.0250586.
