# Supplementary figures and images for: Nutritional markers and proteome in patients undergoing treatment for pulmonary tuberculosis differ by geographic region
Source: PLoS One. 2021 May 5;16(5):e0250586. doi: 10.1371/journal.pone.0250586 (PMC8099102; doi:10.1371/journal.pone.0250586)

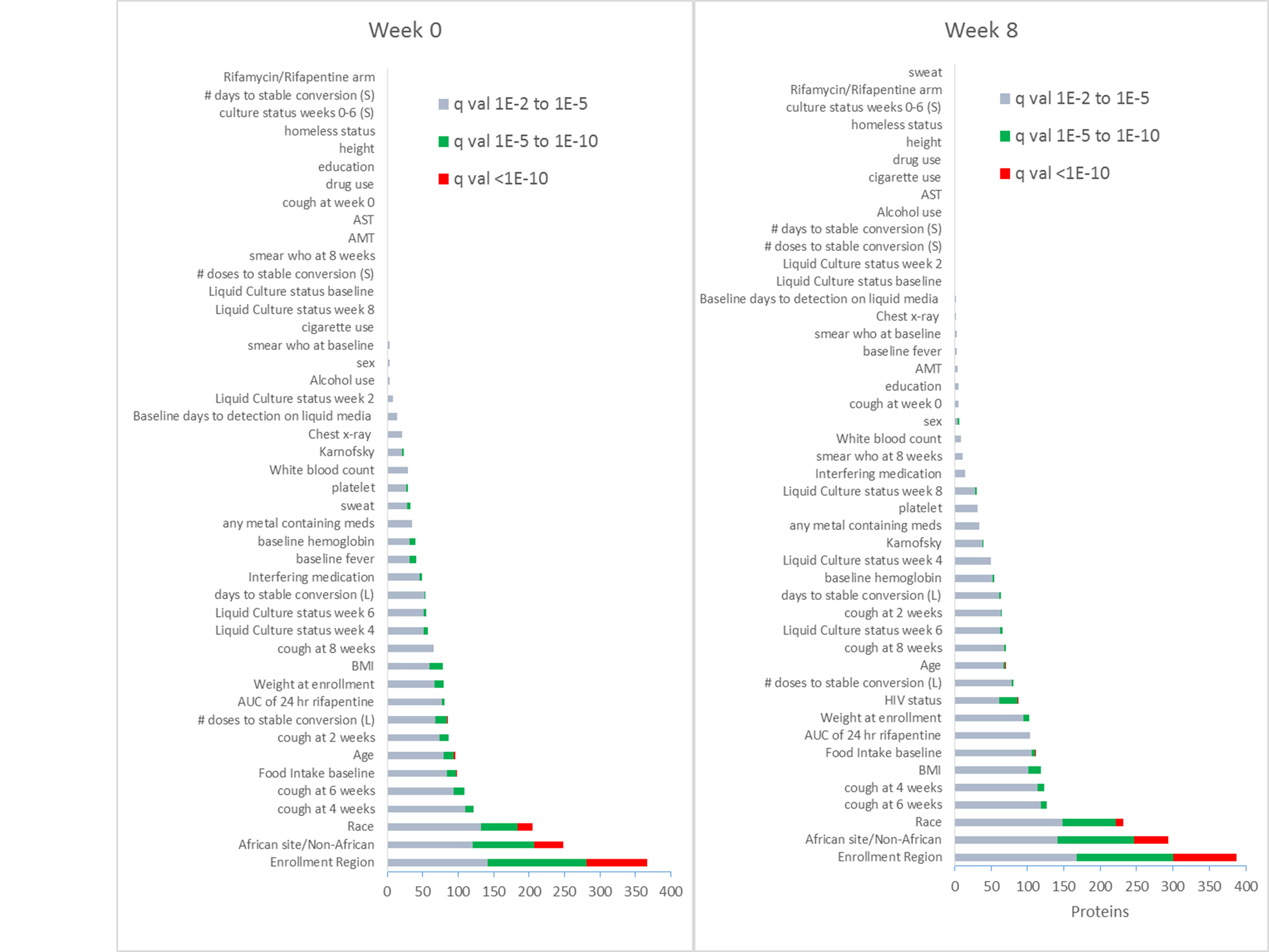

Supplement: S1 Fig — Univariate ANOVA analysis showing the number of proteins which are deemed significantly differential for each clinical covariate at various adjusted p value (q-value) levels. Noted is the most differential covariates of region, African status, and race (all interrelated), which is maintained through 8 weeks of treatment. (TIF) [file pone.0250586.s001.tif]

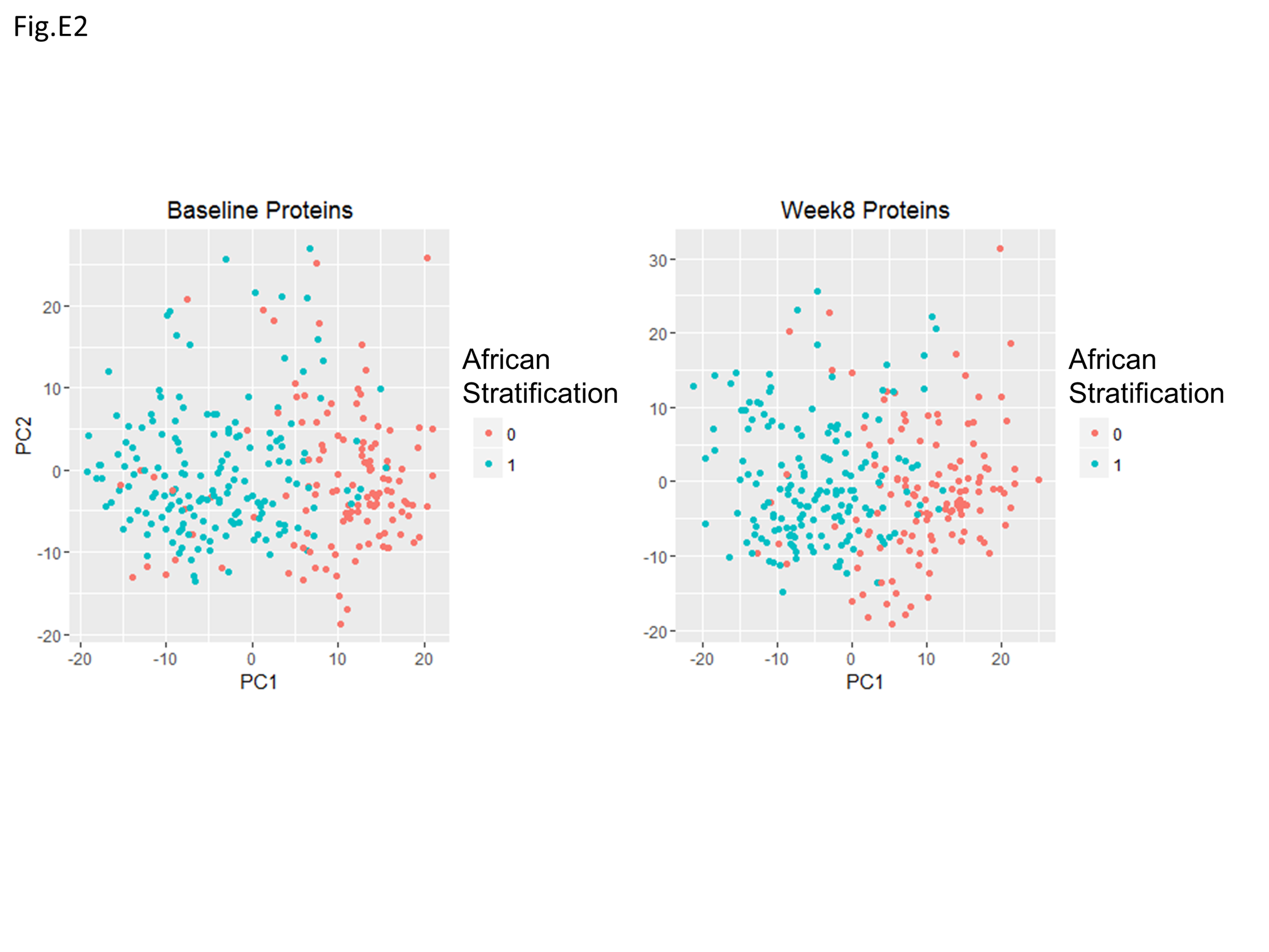

Supplement: S2 Fig — Principle Components Analysis (PCA) plot showing stratification of all TB patients based on African/non-African enrollment region. Blue representing African patients, and red representing non-African patients. (TIF) [file pone.0250586.s002.tif]

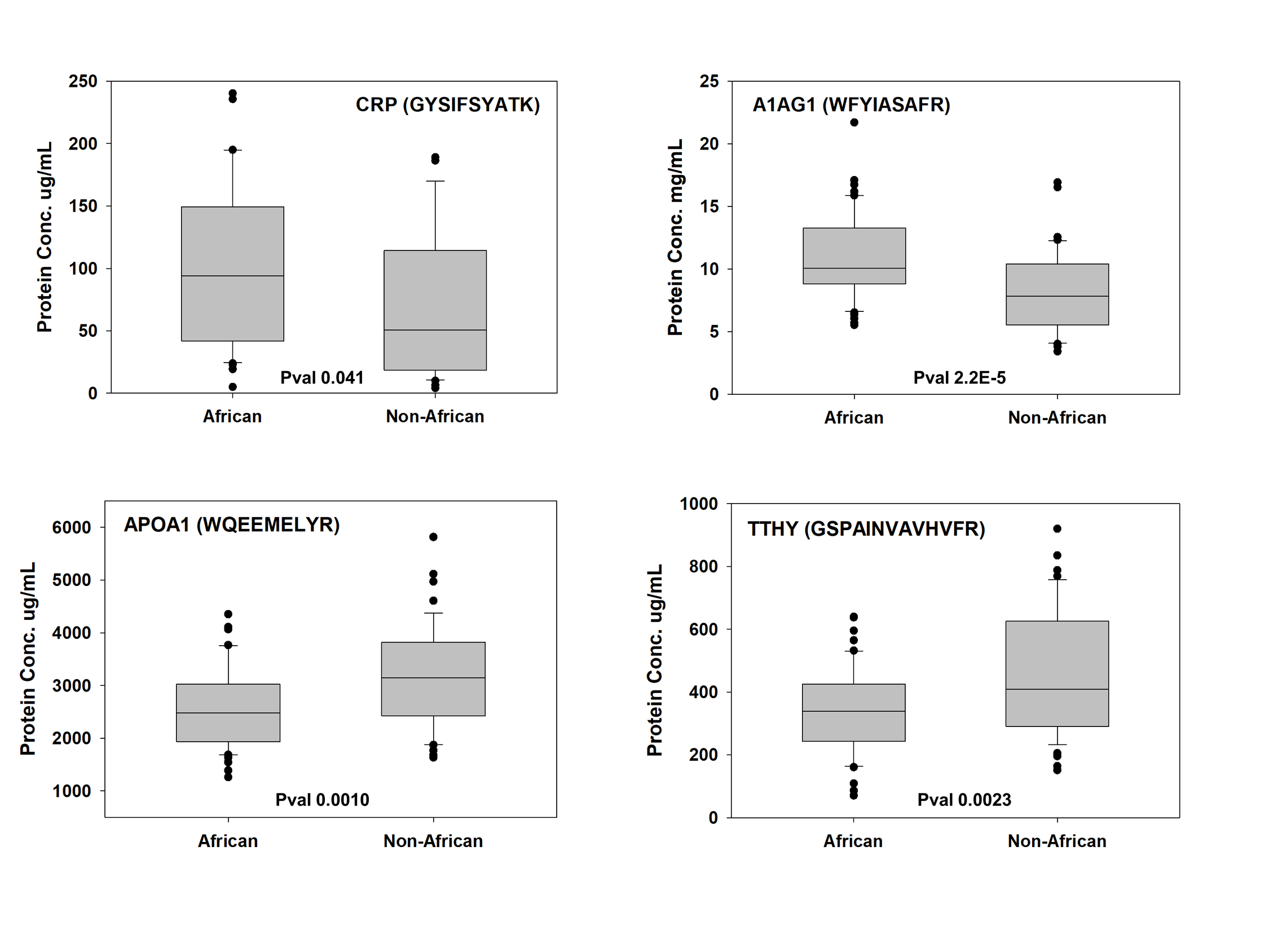

Supplement: S3 Fig — Boxplot representation of Selective Reaction Monitoring (SRM) MS data of specific differential proteins to validate the previous global quantitative data. The peptide sequence which was used as the accurate quantitative internal standard is given for each protein. Two-tailed t-test p-values are provided for each comparison, which all show significance at the <0.05 confidence level. (TIF) [file pone.0250586.s003.tif]

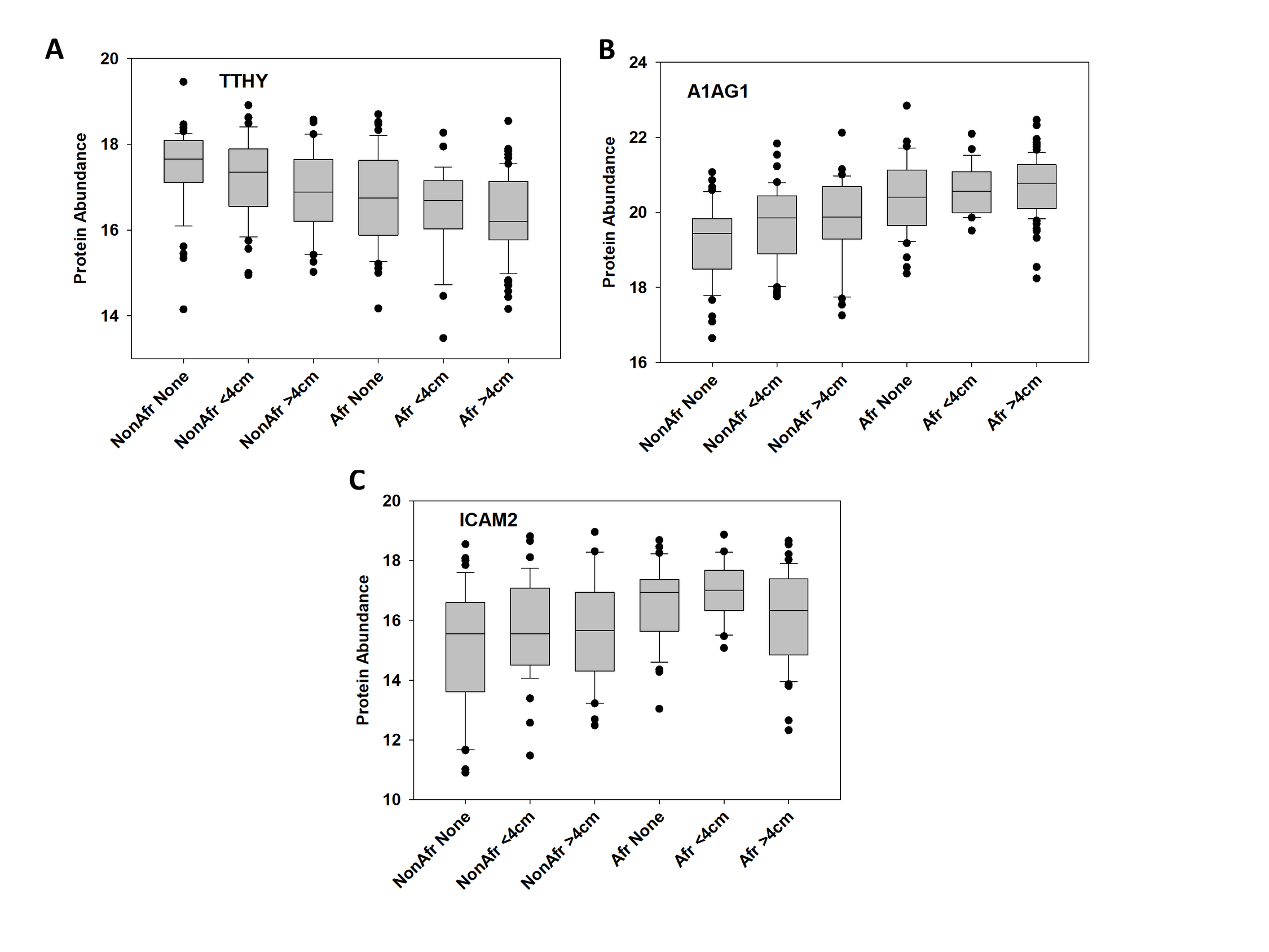

Supplement: S4 Fig — A-C) Boxplot representation of quantitative global MS data for three additional relevant proteins representative of inflammatory, host response, and immune activation showing both up and down regulation within the African cohort stratified by cavitary size. (TIF) [file pone.0250586.s004.tif]
